# Cognitive enrichment improves spatial memory and alters hippocampal synaptic connectivity in a mouse model for early-life stress

**DOI:** 10.3389/fncel.2025.1646883

**Published:** 2025-10-17

**Authors:** Justin L. Shobe, Elham Ghanbarian, Robert Bain, Rajat Saxena, Meenakshi Chandrasekaran, Bruce L. McNaughton

**Affiliations:** 1 Department of Neurobiology and Behavior, University of California, Irvine, CA, United States; 2 Department of Neuroscience, Canadian Centre for Behavioral Neuroscience, University of Lethbridge, Lethbridge, AB, Canada

**Keywords:** memory, synaptic plasticity, behavior, hippocampus, enrichment, early-life stress and mossy fiber

## Abstract

Early-life stress (ELS) and enrichment often have opposing effects on long-term cognitive abilities. Deprivation, such as institutionalized care during early childhood neurodevelopmental periods, results in lifelong working memory and recall deficits. In contrast, enrichment facilitates new learning and slows cognitive decline due to aging and neurodegenerative diseases. Similarly, in rodent models, enrichment facilitates learning whereas ELS induces prominent spatial memory deficits. Environmental enrichment (EE) and ELS can cause opposing changes in hippocampal structure (e.g., shifts in synaptic density) that largely depend on experimental conditions. However, it remains untested whether EE can rescue the behavioral disruptions caused by ELS and how this would impact the hippocampus at advanced ages. To address this, we conducted a longitudinal study on ELS mice, extensively training them on a cognitive enrichment track (ET) or an exercise alone control track (CT). After this, the mice underwent repeated memory testing followed by brain extraction for anatomical analysis of their hippocampus. We found that ET reversed spatial memory deficits at 6, 13, and 20 months and reduced the number of dentate gyrus (DG) to CA3 synapses. Surprisingly, this reduction occurred at excitatory MF synapses surrounding CA3 somas in the stratum pyramidale—a layer not typically associated with MF terminals. Collectively, these findings suggest that cognitive enrichment during early adulthood may reverse ELS-induced spatial memory deficits by adjusting synaptic connectivity between the DG and CA3.

## Introduction

Severe early-life adversity, affecting close to 50% of the world’s children (Fenoglio et al., 2006), can cause both emotional and cognitive disturbances. For instance, children raised in institutionalized settings often exhibit memory deficits and poor impulse control (Pollak et al., 2010); likely contributing to their delayed language acquisition and low scholastic aptitude (Beckett et al., 2007; Eigsti et al., 2011). Evidence suggests that early childhood deprivation, such as parental neglect, has a particularly negative impact on executive function and memory (Wodarski et al., 1990; Eckenrode et al., 1993; Spratt et al., 2012) because this sensitive time period (first 2–3 years in humans and first 3–4 weeks in rodents) is critical for the maturation of brain systems necessary for learning and memory, such as the hippocampus (Malave et al., 2022; Kloc et al., 2020; Leinekugel et al., 2002). This may be why ELS during this time period can cause long-lasting deficits in declarative learning and hippocampal function. Adults who have experienced ELS have difficulty remembering episodic events and perform poorly on delayed word recall tasks (Ding and He, 2021; Ma et al., 2021; Cai et al., 2024). These individuals have smaller hippocampal volumes, especially their dentate gyrus (DG) (Humphreys et al., 2019; Youssef et al., 2019; Koyama et al., 2022; Kawamoto et al., 2023). Importantly, these findings have been replicated in rodent models designed to mimic maternal neglect (Walker et al., 2017). As adults, these ELS animals perform poorly on hippocampus-dependent spatial memory tasks such as the Morris water maze and object location memory (OLM) (Brunson et al., 2005; Cui et al., 2006; Rice et al., 2008; Ivy et al., 2010; Pollak et al., 2010; Naninck et al., 2015; Chen et al., 2016; Molet et al., 2016b; Naninck et al., 2017) consistent with human findings, these animals have smaller hippocampi and dendritic atrophy in CA3 and CA1 neurons (Brunson et al., 2005; Molet et al., 2016a; Teicher et al., 2016).

Unfortunately, there are very few non-invasive treatments for individuals suffering from the effects of ELS; however, experience-based behavioral interventions may help. For example, the Bucharest intervention project (and related studies) found that adoption of institutionalized children into more nurturing environments correlated with improved cognition and higher IQ scores (i.e., the earlier the better) (Tizard and Rees, 1974; Nelson et al., 2007; Bos et al., 2009; Almas et al., 2016). And, enriching activities later in life can also facilitate cognitive recovery (Cai et al., 2024).

In rodent models, environmental enrichment (EE) improves cognitive functions, including spatial learning, memory, and task learning (Cheng et al., 2022; Rountree-Harrison et al., 2018; Bennett et al., 2006; Williams et al., 2001; Woodcock and Richardson, 2000; Zeleznikow-Johnston et al., 2017). Consistent with this, we found that extensive cognitive enrichment on a specially designed “enrichment track” leads to dramatic improvements on a wide variety of memory tasks (Gattas et al., 2022). Mechanistically, EE promotes synaptogenesis and adult hippocampal neurogenesis (Speisman et al., 2013) increases spine count (Jung and Herms, 2014) and dendritic complexity (Connor et al., 1982), modulates synaptic signaling (Pintori et al., 2024) and enhances long-term potentiation in the hippocampus (Cortese et al., 2018; Artola et al., 2006). Additionally, it improves sensory processing and neural coding efficiency (Engineer et al., 2004; LeMessurier et al., 2019). For instance, EE improves the ability of the hippocampus to distinguish between different environments (i.e., better pattern separation), which in turn promotes better spatial learning (Bilkey et al., 2017; Ventura et al., 2024).

The DG-CA3 connection is particularly susceptible to experience-dependent plasticity (Henze et al., 2000; Urban et al., 2001). ELS and EE can have opposing effects on this such as the rate of neurogenesis in the DG and the magnitude of DG-CA3 long-term plasticity (LTP and LTD) (Alwis and Rajan, 2014; Kempermann, 2019). In contrast, both ELS and EE increase mossy fiber sprouting (Brunson et al., 2001; Galimberti et al., 2006; Bramati et al., 2023). This paradoxical finding—that both detrimental ELS and beneficial EE increase mossy fiber sprouting—highlights the need to examine not just the quantity but also the location and functional properties of these synaptic changes. This is especially interesting, considering the positive correlation between mossy fiber expansion and spatial memory performance (Schwegler et al., 1990; Ramírez-Amaya et al., 2001; Carasatorre et al., 2015). With respect to EE, however, it is important to recognize that many of these structural and physiological changes largely depend on experimental conditions such as the animal’s age and the duration of enrichment (Gogolla et al., 2009; Bednarek and Caroni, 2011); making it difficult to predict how ELA and EE would mechanistically interact.

Despite extensive research on ELS and EE individually, critical knowledge gaps remain. First, while EE has been shown to enhance cognition in normal animals, it is unknown whether cognitive enrichment can reverse established ELS-induced memory deficits, particularly across the lifespan. Second, although both ELS and EE affect hippocampal structure, the specific synaptic mechanisms underlying potential ELS-EE interactions remain unexplored. Finally, previous studies have not distinguished between the effects of cognitive enrichment versus exercise alone in the context of ELS recovery, limiting our understanding of which intervention components are most therapeutic.

We hypothesized that cognitive enrichment would reverse ELS-induced spatial memory deficits across the lifespan through specific modifications to mossy fiber synaptic connectivity between the dentate gyrus and CA3 region. To test this hypothesis, we pursued three specific objectives: (1) determine whether cognitive enrichment can reverse ELS-induced spatial memory deficits longitudinally from young adulthood through aging; (2) distinguish the effects of cognitive enrichment from exercise alone using a controlled track design; and (3) identify the underlying synaptic mechanisms by examining mossy fiber terminal density and size distribution in the hippocampus.

Using a longitudinal approach, we first trained ELS mice on our complex obstacle enrichment track (ET) (Supplementary Figures 1A, B). This 3-month protocol produces dramatic and broad long-term memory enhancements well above that of standard enrichment procedures (Gattas et al., 2022). Moreover, the incorporation of a simple ramp control track (CT) group allowed us to disambiguate the effect of exercise alone. Following this, we repeatedly tested mice on spatial and object recognition tasks across their lifespan. Finally, we assessed whether these transgenic mice (GCaMP6fs) had hippocampal structural changes using a combination of endogenous fluorescence and nanobodies. To specifically visualize MF projections and their synapses, we took advantage of the selective expression of the GCaMP6fs signal in the DG (no signal in CA3 neurons) together with excitatory synaptic markers such as PSD-95, which are primarily considered glutamatergic (Walker et al., 2001; Gutiérrez et al., 2003); however, there is some evidence that these terminals also contain GABA (Cabezas et al., 2012; Uchigashima et al., 2007). This approach allowed us to test whether cognitive enrichment could rescue ELS deficits and identify the specific synaptic adaptations underlying any behavioral recovery. Unexpectedly, we found clusters of these atypical excitatory MF synapses surrounding CA3 cell bodies whose density was regulated by the cognitive component of enrichment.

## Results

### Cognitive enrichment rescues the spatial memory deficits of ELS mice

**FIGURE 1:**
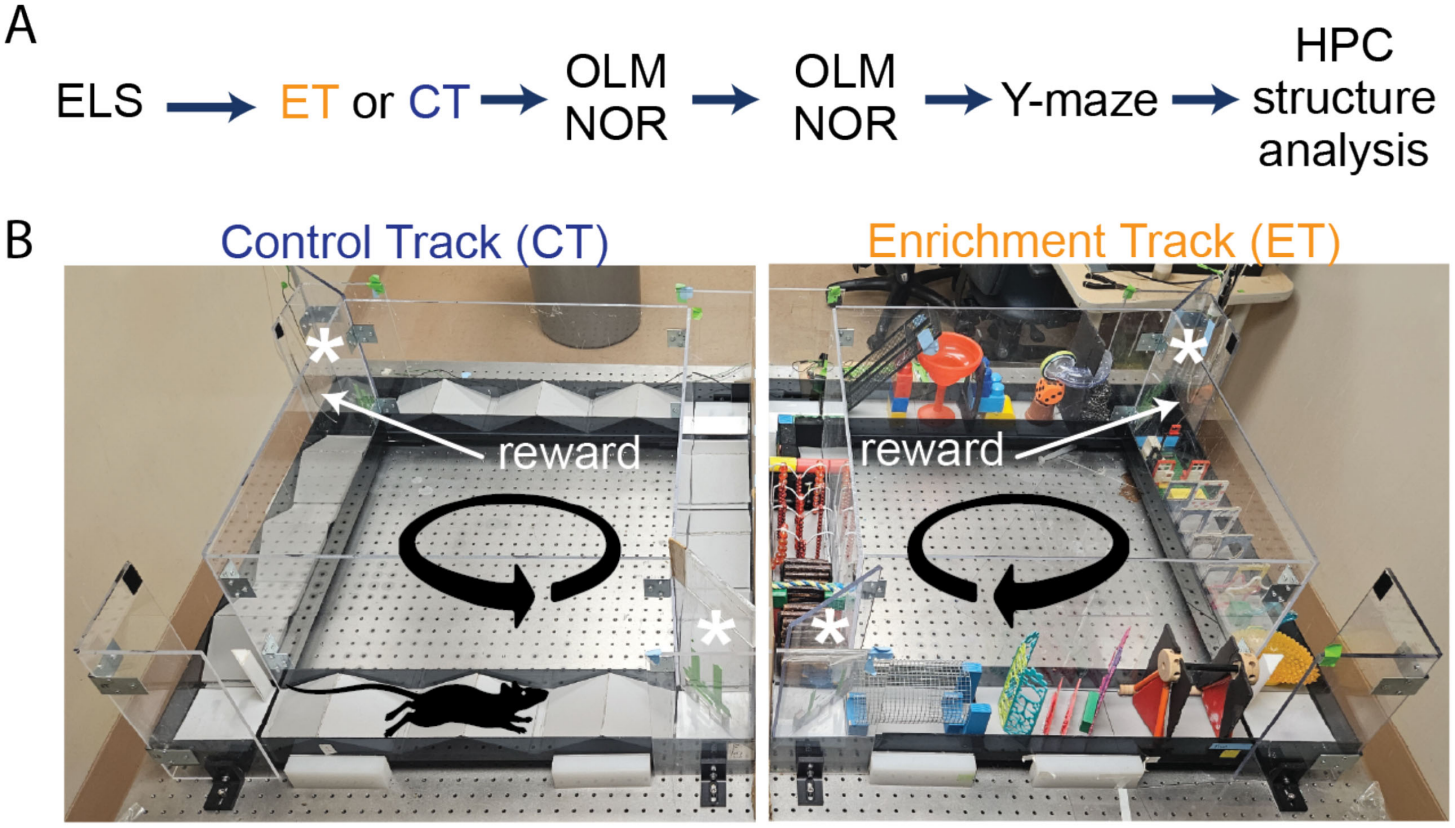
Experimental timeline of enrichment training, behavioral tests and structural analysis on early-life stressed (ELS) and control mice. **(A)** Initially, we separated ELS mice into two sex matched groups. Half of the ELS mice (four males and four females) ran on the enrichment track (ET) track (orange) while the other half (four males and four females) ran on the control track (CT) track (dark blue). Following this, both groups received longitudinal behavioral testing and hippocampal structural analysis. **(B)** We ran double-housed adult ELS mice on our automated side-by-side environmental enrichment (EE) setups such that one mouse ran on the control track (CT, left panel) while its cage-mate ran on the enrichment track (ET, right panel). On the ET track, mice had to navigate through multiple obstacles (obstacle configuration changed every session), whereas the CT only ran over simple ramps. We place two one-way doors (asterisks) at opposite corners of the square track to minimize backtracking. Mice received a reward (sweetened condensed milk) upon completion of each lap controlled by an automated delivery system using an overhead camera tracking system.

First, we induced ELS using the disrupted maternal care paradigm (limited bedding and nesting method, LBN) since it causes progressive memory deficits as rodents age (Molet et al., 2016a; Rocha et al., 2021). Following this we assigned mice to either enrichment or control groups (Figure 1A). Our previous findings suggest that ET training produces broad and dramatic memory enhancements (Gattas et al., 2022). We speculated, however, that a scaled back version of the ET training protocol would more selectively benefit hippocampal circuits that are especially vulnerable to ELS (Scholz et al., 2015; Molet et al., 2016b; Hoeijmakers et al., 2018; Manno et al., 2022). Thus, we ran our enrichment protocol for three 30 min sessions per week, one quarter of our original six 1 h sessions per week.

**FIGURE 2:**
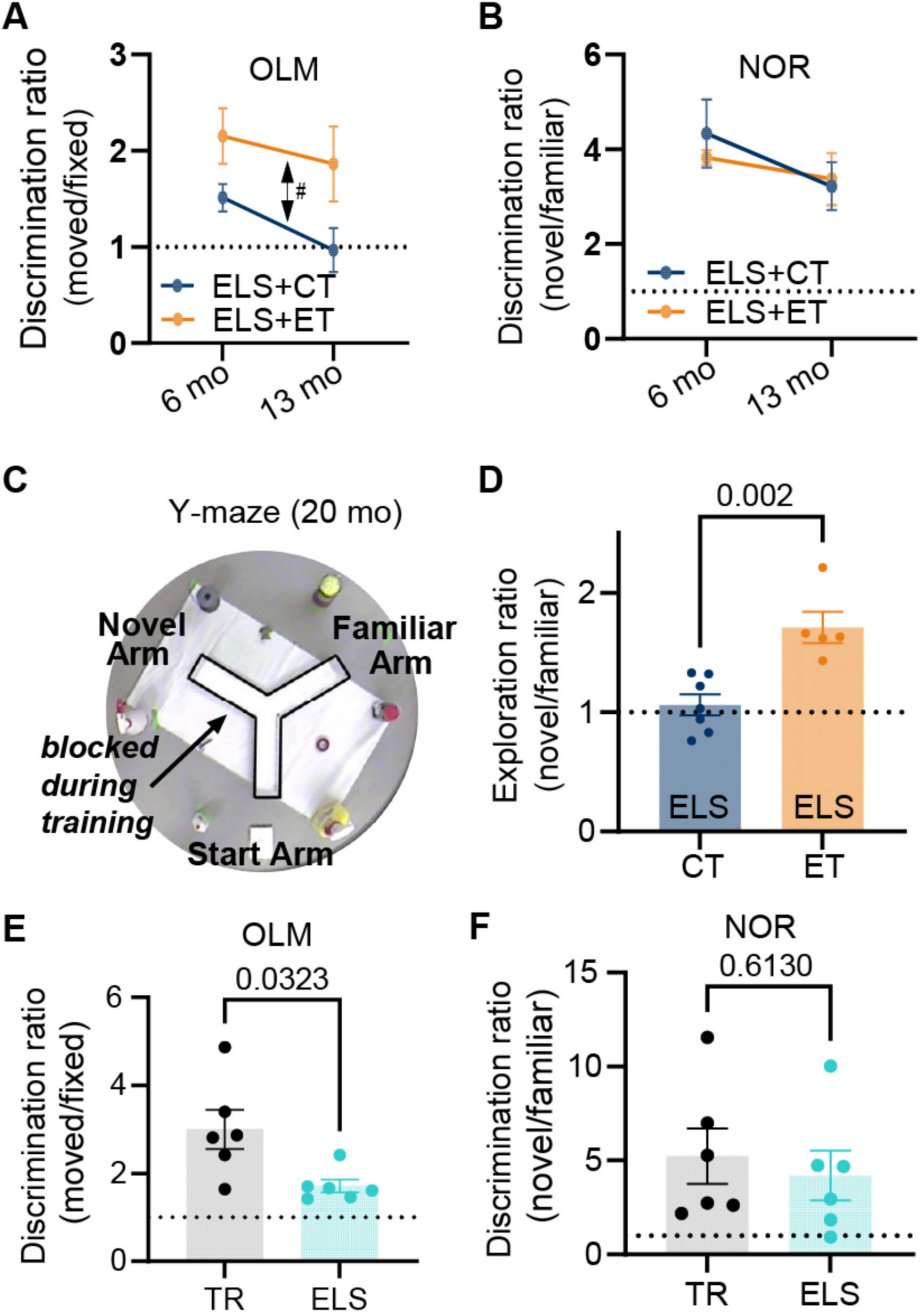
Enrichment track (ET) training reverses life-long spatial memory deficits caused by early-life stress (ELS). Longitudinal testing of ELS mice on object-location (OLM) and novel object recognition (NOR) at mature (6 months) and middle-age (13 months) time points revealed that. **(A)** The ELS + ET group (orange, *n* = 8 mice) had improved 24 h memory [double-sided arrow, three-way repeated measures ANOVA, F(1,12) = 8.80, *p* = 0.012] for OLM relative to the ELS + CT control group (dark blue, *n* = 8). There was no significant effect of age or sex. **(B)** In contrast for NOR, we found no group difference [F(1,12) = 0.074, *p* = 0.791] and no effect of age or sex. The same mice (*n* = 16) were tested on OLM and NOR. A value of 1 represents equal time with both objects (dashed line). **(C)** In a final (20 months) Y-maze test, the surviving mice (5M and 7F, *n* = 12) explored the two open arms for 5 min while the third arm remained blocked (transparent walls surrounded by distinct external cues) followed 24 h later by testing (5 min session) where mice were allowed to freely explore all three arms. **(D)** The ELS + ET group (orange, *n* = 5) spent significantly more time exploring the novel arm in comparison to the ELS + CT group (dark blue, *n* = 7) [two-way ANOVA, F(1,8) = 19.89, *p* = 0.002]. We calculated the active exploration ratios by dividing the occupancy times of the novel arm by that of the familiar arm (we excluded periods of immobility lasting longer than 1 s). **(E,F)** To verify that ELS alone (in the absence of EE) induced memory deficits, we tested mice on OLM and NOR using the same conditions as group 1. **(E)** In OLM, the TR group (black dots, *n* = 6 males) had significantly higher discrimination ratios (moved/fixed) compared to the ELS group (cyan dots, *n* = 6 males), indicating that they spent more relative time investigating the moved object (*p* = 0.0323, unpaired two-tailed *t*-test with Welch’s correction). **(F)** For NOR we observed that no significant difference in discrimination ratios between the same TR and ELS groups (*p* = 0.613, unpaired two-tailed *t*-test). Plots include values from individual mice [circles in **(D–F)**], mean [bar height and circles in **(A,B)**], and SEM (error bars).

We then tested both groups (ELS + ET, 4M and 4F, *n* = 8) and (ELS + CT, 4M and 4F, *n* = 8) on OLM and NOR memory tests at mature (6 months), middle (13 months) and old (20 months) ages to determine if behavioral gains would last as the mice aged (Figures 1A, B). We observed a significant group level (ET vs. CT) difference in OLM [three-way ANOVA, F(1,12) = 8.80, *p* = 0.012] but no significant effect of age [F(1,12) = 1.69, *p* = 0.218] or sex [F(1,12) = 0.97, *p* = 0.345] (Figure 2A). In contrast to OLM and also the results of Gattas et al. (2022), we found no significant group [three-way ANOVA, F(1,12) = 0.074, *p* = 0.791], age [F(1,12) = 2.93, *p* = 0.113], or sex [F(1,12) = 0.11, *p* = 0.744] effect in the NOR test (Figure 2B). In the final OLM/NOR time-point (18 months), the mice, unfortunately, did not spend enough time exploring the objects to get accurate testing scores (values are the sum of investigation times for both objects). Compared to their mature and middle-age scores, these older mice spent significantly less time investigating the objects [Supplementary Figure 2B, two-way ANOVA, *P* < 0.0001, F(2,88) = 30.86]; likely due to habituation to the OLM/NOR setup, an age-dependent reduction in exploratory behavior, or a combination of both. To reinvigorate their exploratory behavior, we switched to a completely novel setup, the spatial Y-maze. On test day, we found that ET mice (2M &3F, *n* = 5) spent significantly more time exploring the novel arm than CT mice (3M &4F, *n* = 7) in a group comparison [two-way ANOVA, F(1,8) = 19.89, *p* = 0.002], with no significant effect of sex [F(1,8) = 1.81, *p* = 0.215], suggesting that ET produced a life-long rescue of spatial memory formation or retention (Figures 2C, D).

Importantly, our design mitigates the exercise confound since both groups must run laps to receive rewards. In fact, the control track (CT) group, that ran over simple ramps, completed more laps than the ET group that had to navigate through complex obstacles (Supplementary Figure 1C, unpaired two-tailed *t*-test, *p* < 0.0001). Thus, additional exercise cannot account for these observed memory enhancements in the ET group.

### ELS causes spatial memory deficits

Many studies have shown that ELS causes significant memory disruptions (Brunson et al., 2005; Cui et al., 2006; Rice et al., 2008; Ivy et al., 2010; Wang et al., 2011; Naninck et al., 2015, 2017; Molet et al., 2016a; Hoeijmakers et al., 2018). To confirm these findings, we tested a separate non-enriched group of male mice under either ELS or typical rearing (TR) conditions (Figure 1A, chartreuse arrow). These ELS mice and their age-matched TR controls remained under normal housing conditions until behavioral testing at mature adult ages (∼6 months). The ELS group (6 months) showed memory impairments in the OLM task (*p* = 0.032, two-tailed *t*-test, Figure 2E bottom) but not in NOR (*p* = 0.613, Figure 2F bottom). Taken together, our findings are consistent with other studies that ELS can cause a selective disruption of hippocampal -dependent memories (Molet et al., 2016b; Hoeijmakers et al., 2018) but also see (Ivy et al., 2010; Naninck et al., 2015).

### Cognitive enrichment does not change gross hippocampal morphology

**FIGURE 3:**
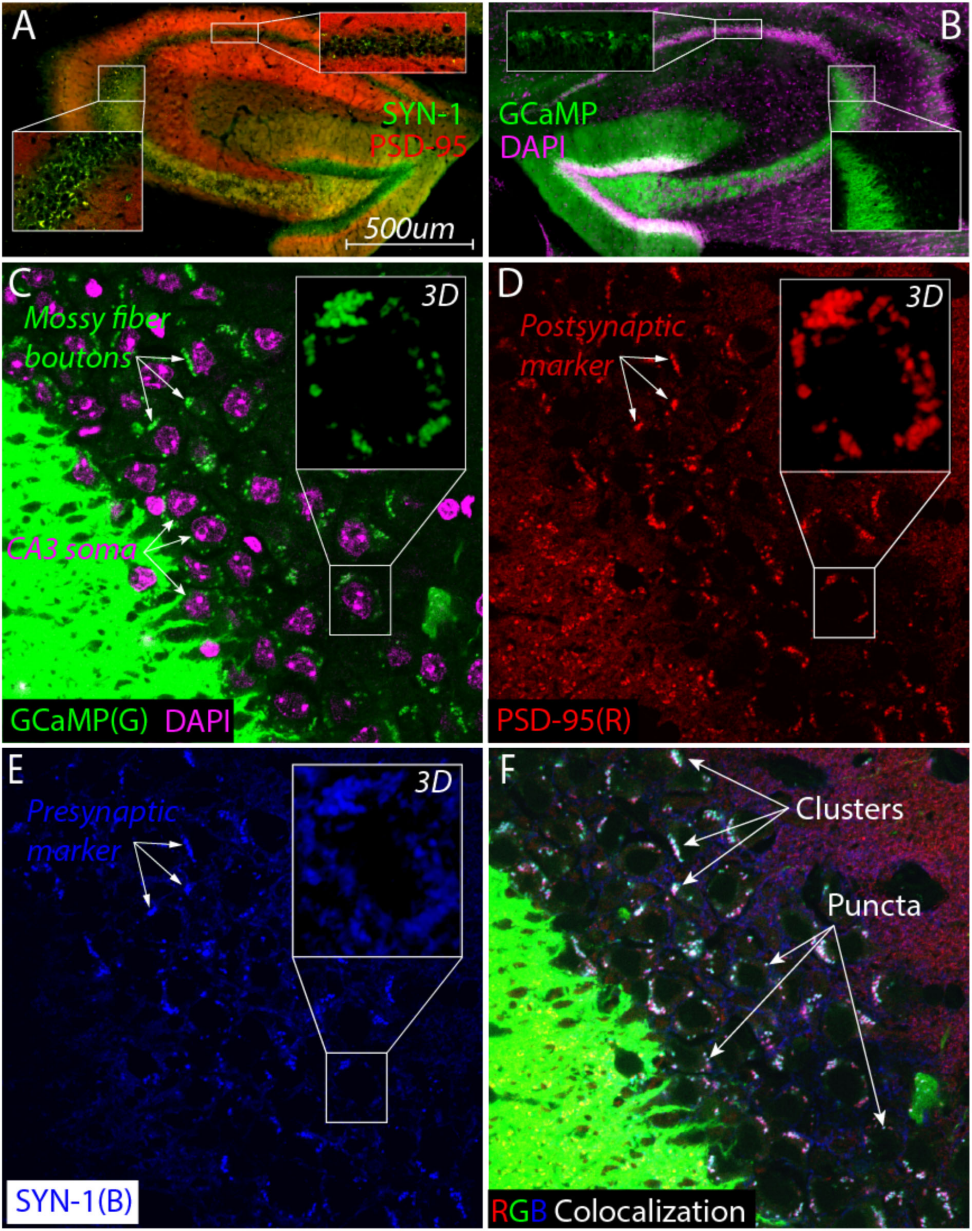
Large synaptic clusters containing mossy fiber boutons surround CA3 cell bodies in aged ELS mice (20 months). **(A)** Colocalization of postsynaptic marker αPSD-95 nanobody (red) with presynaptic marker αSynaptotagmin nanobody (SYN1, green) reveals the presence of large synaptic clusters (yellow) around CA3 soma (bottom inset) but not in the CA1 pyramidal subfield (top inset). **(B)** High expression of GCaMP signal (green) in the dentate gyrus cells show that mossy fibers project and appear to terminate near CA3 cell bodies (purple, DAPI). GCaMP signal is not detectable inside CA3 neurons (right inset) but it does fill a large percentage CA1 pyramidal neurons (top inset) as expected from imaging studies. **(C–F)** Super resolution single-plane optical section imaging of the stratum pyramidale [**(C)**, CA3 cell bodies labeled with DAPI, purple] stained for mossy fiber boutons [**(C)**, green], postsynaptic PSD-95 [**(D)**, red] and presynaptic SYN-1 [**(E)**, blue] yields extensive triple colocalization [**(F)**, white] that consists of larger clusters and smaller individual puncta consistent with the hallmarks of MF synapses. Max Z projections of zoomed in z-stack images from a single cell body [**(C–E)**, insets] demonstrate that these synaptic clusters surround the cell in all three dimensions.

Studies have shown that enrichment and ELS can cause volumetric changes in hippocampal subfields such as the mossy fiber (MF) pathway (Brunson et al., 2005; Galimberti et al., 2006; Molet et al., 2016b; Teicher et al., 2016; Humphreys et al., 2019; Youssef et al., 2019; Bramati et al., 2023). This prompted us to examine whether these ELS GCaMP-expressing mice mice had any gross morphological changes in their hippocampal structure. We used Thy1- because our earlier pilot images revealed that their DG neurons and axons (the MF pathway) were filled with GCaMP signal. One month following the final Y-maze test (21 months of age), we extracted brains for fluorescent imaging of endogenous GCaMP and DAPI signals in the dorsal hippocampus. Importantly, this GCaMP signal was absent from CA3 neurons, indicating that the GCaMP signal in the CA3 subfield was specific to MFs (Figure 3B lower right inset). As expected, there was sparse GCaMP expression in CA1 neurons (Figure 3B top inset).

Given the specificity of our GCaMP signal in combination with DAPI staining, we measured the areas of the GC Layer, the Hilus, and the suprapyramidal blade of the MF. After normalization to total hippocampal area (Supplementary Figure 4A), we did not detect any significant differences in these areas between ELS + ET (*n* = 5) and ELS + CT (*n* = 7) mice (Supplementary Figure 4B). The number of CA3 neurons per 0.01 mm^2^ was not significantly different between groups. Altogether, this suggests that ET training does not cause gross structural changes in the DG to CA3 circuit when compared to our exercise alone control group (CT).

#### Identification of atypical MF synapses surrounding CA3 cell bodies

To visualize synapses, we found it necessary to use nanobodies that are capable of penetrating fixed tissue (Kilisch et al., 2023) allowing us to compare presynaptic and postsynaptic markers as well as the endogenous GCaMP signal (the MF indicator) in the same slice. Immunostaining with synaptotagmin and PSD-95 revealed the presence of prominent large synaptic clusters surrounding the CA3 soma that were not as visible in other areas such as the CA1 subfield (Figure 3A top inset).

The large synaptic clusters, consistent with MF boutons, were particularly notable in the CA3 stratum pyramidale, which was unexpected. To confirm these were bona fide MF synapses, we individually visualized all three signals: mossy fiber GCaMP (Figure 3C, green), postsynaptic PSD-95 (Figure 3D, red), and presynaptic synaptotagmin (Figure 3E, blue). Around CA3 cells (purple staining in Figure 3C), we observed many clusters in the same location across all three images (white arrows in Figures 3C–E). The observed putative MF boutons colocalized with PSD-95 (Figures 3C, D) as well as VGLUT1 (Supplementary Figure 5) suggesting that they were excitatory synapses.

The triple colocalization image (overlay of all three RGB colors) revealed that many clusters were white, suggesting they were composed of all three markers (Figure 3F). These clusters varied in size, from larger clusters (top three arrows, Figure 3F) to individual puncta (bottom three arrows, Figure 3F). We performed a 2D spatial cross-correlation on these images to statistically determine the extent of colocalization. A pairwise analysis of all three combinations (PSD95-SYN1, PSD95-GCaMP, and SYN1-GCaMP) revealed significant overlap, with peaks higher than the shuffled distribution (Sup Fig. 3). This statistically significant triple colocalization of mossy fiber boutons and pre- and postsynaptic markers strongly supports the presence of excitatory MF synapses in the CA3 pyramidal layer.

### Characterization of atypical MF synapses in the SP layer

**FIGURE 4:**
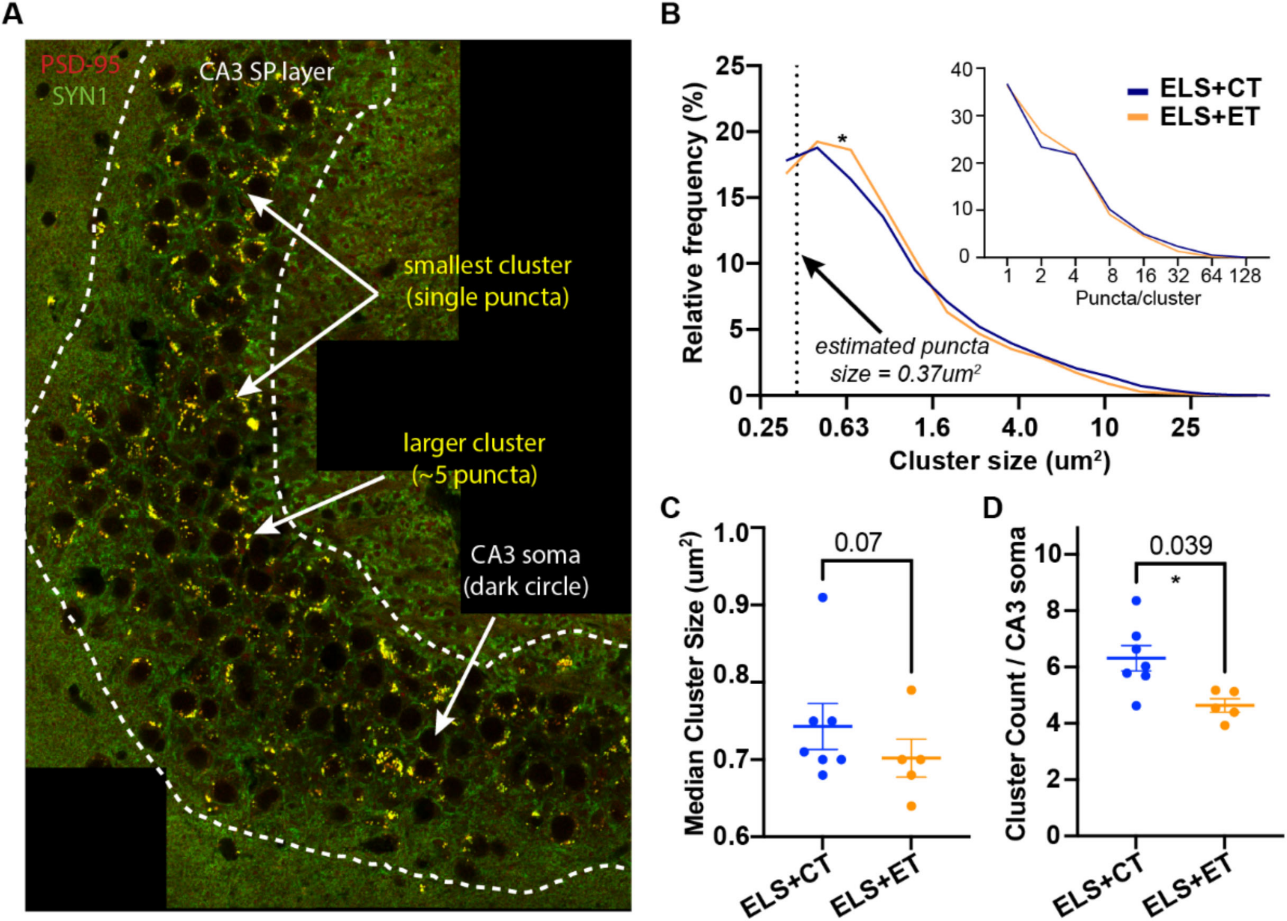
Enrichment track (ET) training reduces the density of putative mossy fiber (MF) synapses in the CA3. **(A)** A representative tiled image from an ELS + ET mouse (brain harvested following Y-maze) double-stained with PSD95 (red) and SYN1 (green) nanobodies. In the stratum pyramidale (SP) region (dashed white line) of the CA3, colocalized (yellow) clusters appear to be comprised of individual puncta (area = ∼200 pixels or 0.37 μm^2^). Clusters smaller than 150 pixels were removed from subsequent analysis. **(B)** Histogram of log-transformed cluster sizes (pooled) and estimated number of puncta/cluster (inset) identified from ELS + ET (10,497 clusters, *n* = 7 mice) and ELS + CT mice (5,847 clusters, *n* = 5 mice). Statistical analysis between the groups using rank-based (Mann Whitney, *p* = 0.035) and cumulative (Kolmogorov-Smirnov, *p* = 0.0001) methods indicate significant differences (asterisk) in their distributions, likely due to fewer puncta per cluster in ELS + ET mice (inset). Dashed line indicates the x-intercept corresponding to our estimated size of a single puncta. **(C,D)** A mouse-by-mouse analysis reveals that ELS + ET mice (*n* = 5, orange dots) have fewer clusters compared to ELS + CT controls (*n* = 7, dark blue dots, two-way ANOVA, F(1,8) = 6.07, *p* = 0.039, asterisk, **(D)**. There was a trend for smaller median cluster sizes in the ELS + ET group (two-way ANOVA, F(1,8) = 4.36, *p* = 0.070, **(C)**. Tiled sections were normalized to counts to the total number of CA3 neurons.

Next, we examined quantitative differences in putative excitatory MF synapses between the ELS + ET and ELS + CT mice. We immunostained sections for postsynaptic PSD-95 (red) and presynaptic synaptotagmin 1 (SYN1, green) as this combination of synaptic markers had the highest overlap score (see the green line, Supplementary Figure 3). We tiled a large area of the CA3 bend to identify MF synapses (yellow clusters and white arrows) of various sizes as well as CA3 cell bodies (dark circles) (Figure 4A). Regardless of size, colocalized clusters in the stratum pyramidale (SP) region (dashed white line) of the CA3, appeared to be composed of individual puncta with an area = ∼200 pixels or 0.37 μm^2^ (Figure 4A).

We used automated counting to determine frequency and size of clusters in the tiled images (2 per mice) between ELS + ET and ELS + CT mice. In total, ELS + ET mice had 10,497 clusters (*n* = 7 mice) while ELS + CT mice had 5,847 clusters (*n* = 5 mice). Statistical analysis between the pooled groups using rank-based (Mann-Whitney, *p* = 0.035) and cumulative (Kolmogorov-Smirnov, *p* = 0.0001) methods indicated significant differences in their size distributions. ELS + ET mice (orange line) had fewer large clusters and proportionally more smaller clusters than ELS + CT controls (blue line, Figure 4B). ELS + ET mice had a corresponding shift in puncta per cluster in (Figure 4B inset); assuming that the larger clusters are composed of discrete puncta.

In a mouse-by-mouse analysis, normalized per CA3 neuron, we found that ELS + ET mice had on average 27% fewer clusters (4.75 ± 0.15, *n* = 5, orange dots) compared to ELS + CT controls (6.31 ± 0.63, *n* = 7, dark blue dots, two-way ANOVA, F(1,8) = 6.07, *p* = 0.039) with no significant sex differences [F(1,8) = 0.043, *p* = 0.841 Figure 4D). There was no significant group or sex difference in median cluster size [two-way ANOVA, group: F(1,8) = 4.36, *p* = 0.070; sex: F(1,8) = 0.0009, *p* = 0.976, Figure 4C], although there was a small trend for smaller synapses in ELS + ET mice consistent with our distribution analysis. These findings suggest that ET training reduces the number of mossy fiber (MF) synapses surrounding CA3 somas in ELS mice without significantly affecting the size of individual synaptic clusters.

## Discussion

In a mouse model of ELS, we found that cognitive enrichment training during young adulthood leads to long-lasting changes in spatial memory and hippocampal structure. To our knowledge this is the first demonstration of these effects in a rodent model of maternal neglect. Compared to exercise alone controls (ELS + CT), mice that underwent enrichment training (ELS + ET) showed significant improvements in long-term OLM (24 h) memory at both mature (6 months) and middle-ages (13 months). Additionally, at 20 months, ELS + ET mice performed better memory in a final spatial Y-maze test. However, we found no group differences in NOR, a task which relies less on the hippocampus (Oliveira et al., 2010; Barker and Warburton, 2011; Cohen and Stackman, 2015). Surprisingly, our anatomical analysis of the hippocampus revealed that aged ELS mice had prominent MF-associated excitatory clusters surrounding the CA3 neurons, an area not typically associated with MF synapses. Furthermore, we found that in aged ELS mice early enrichment reduced the number of these atypical MF synapses by ∼25%.

An important caveat in our findings is that within the stratum pyramidale, we cannot distinguish between atypical excitatory mossy fiber boutons that synapse onto pyramidal cells versus those that synapse onto inhibitory interneurons (Henze et al., 2000; Urban et al., 2001). However, excitatory pyramidal neurons vastly outnumber inhibitory interneurons in the CA3 pyramidal layer, with interneurons comprising only approximately 10%–15% of the total neuronal population (Freund and Buzsáki, 1996; Klausberger and Somogyi, 2008). This numerical predominance suggests that the reduction in excitatory mossy fiber synaptic clusters we quantified likely represents connections onto pyramidal cells. Nevertheless, given that interneurons in this layer provide feed-forward inhibition that regulates CA3 excitability (Jinde et al., 2012), and that environmental enrichment has been shown to modulate inhibitory neurotransmission (Speisman et al., 2013; Cortese et al., 2018), changes in mossy fiber connectivity onto inhibitory neurons could alter CA3 pattern separation. Future studies using cell-type-specific markers or electrophysiological approaches will be necessary to determine whether cognitive enrichment and/or ELS differentially alters these synapses.

There are important similarities and differences between our behavioral observations and those of previous studies. Earlier studies found that ELS can cause wide-ranging spatial (e.g., OLM) and recognition (e.g., NOR) memory impairments (Brunson et al., 2005; Hoeijmakers et al., 2018; Ivy et al., 2010; Molet et al., 2016a; Naninck et al., 2017; Bolton et al., 2020; Short et al., 2020; Rice et al., 2008). These impairments; however, appear to occur at different rates during aging. A careful study, in rats, found that spatial memory deficits start during adolescence whereas the effects of ELS on NOR memory does not occur until older ages (Molet et al., 2016b) [however, see male mice in Naninck et al. (2015) study]. Similarly, we found that our ELS alone mice (6 months) had normal NOR memory but significant deficits in OLM, consistent with the model that the hippocampus is more susceptible to assaults from ELS than other brain regions. Interestingly our observations also suggest the ET protocol has a more selective effect on hippocampus function because between ET and CT mice we found no group differences in NOR. This finding seemingly contrasts with our initial use of the ET protocol, where we found broad memory enhancements (Gattas et al., 2022). The original study; however, used a more extensive protocol (six 1 h sessions/week versus three 30 min sessions/week) that started at an earlier age. This suggests that the abbreviated ET protocol used in this study produces a more limited effect that is biased toward improvements in hippocampal function.

Contrary to our predictions, we found no differences in MF volume between the ELS + ET and ELS + CT groups. Other studies, conducted with non-stressed mice, report that enrichment causes mossy fiber expansion beyond the SL layer (Galimberti et al., 2006; Bramati et al., 2023). Unlike our design, those studies did not control for exercise, as only the EE group had access to a running wheel. Since exercise alone contributes to mossy fiber sprouting (Toscano-Silva et al., 2010), it may be exercise, rather than cognitive enrichment, that drives the growth of mossy fibers toward the CA3 cell bodies.

Several factors could explain why these synapses remain uncharacterized, despite their previous observation (Amaral and Dent, 1981; Dailey et al., 1994; Qin et al., 2001; Banks et al., 2024). Age and ELS cause CA3 dendritic atrophy and mossy fiber sprouting (Brunson et al., 2001; Galimberti et al., 2006; Adams et al., 2010; Molet et al., 2016b), which may be an attempt by the MFs to compensate for synaptic loss, shifting synapse density toward CA3 cell bodies. Another possibility is our use of nanobodies, which are ∼10 times smaller than antibodies. This increases their ability to detect antigens in fixed tissue (Kilisch et al., 2023; Fridy et al., 2024).

How the size and function of these atypical MF synapses compare to that of MF synapses in the SL layer remains unclear. Electron microscopy 3D reconstruction studies estimate the average MF bouton’s cross-sectional area to be ∼5 μm^2^ (Rollenhagen et al., 2007; Rollenhagen and Lübke, 2010; Murray et al., 2020), whereas our 2D average cluster size is ∼1.5 μm^2^. This smaller size could be because our puncta only consist of the synaptic junction (colocalization of pre- and postsynaptic markers) portion of the larger MF structure. Future structural studies with high resolution imaging such as electron microscopy are needed to determine whether these atypical synapses are similar in size to the typical “MF” synapses in the SL layer. Nevertheless, the proximity of these atypical MF synapses to the spike initiation zone suggests a tight coupling between changes in their density and CA3 activity. We suspect that the presence of these synapses would make a significant contribution to the unusually strong property of the MF-CA3 synapses (i.e., “conditional detonator”) as originally proposed by McNaughton and Morris (1987) and theorized by Marr (1971).

The reason why our cognitively enriched mice have fewer MF synapses remains an outstanding question. One possibility is increased synaptic pruning triggered by plasticity such as repeated bouts of long-term depression (LTD) (Bastrikova et al., 2008; Becker et al., 2008; Shinoda et al., 2010). The occurrence of LTD and LTP in the hippocampus largely depends on the nature of the behavioral learning task (Hagena and Manahan-Vaughan, 2024). At MF-CA3 synapses, exposure to a simple novel context facilitates LTP, while introducing large items and rearranging them, even when familiar, facilitates LTD (Kemp and Manahan-Vaughan, 2008; Hagena and Manahan-Vaughan, 2011). Our ET includes large obstacles frequently changed and rearranged, suggesting LTP may occur initially, followed by ongoing LTD and pruning as mice continue learning in the same context.

The fact that MFs from adult-born DG neurons expand beyond the SL layer (Cole et al., 2020) raises the possibility that these atypical synapses preferentially belong to adult-born DG neurons. A recent study found that increases in the sparse activity of CA3 place cells due to enrichment requires neurogenesis (Ventura et al., 2024). EE can also rescue the survival of newborn neurons following ELS (Rule et al., 2021) and newborn neurons are more plastic (Ge et al., 2007; Massa et al., 2011). However, enrichment typically promotes growth factor signaling, enhancing DG-CA3 LTP and MF synaptogenesis (Gogolla et al., 2009; Bednarek and Caroni, 2011; Schildt et al., 2013; Cao et al., 2014). And, whether adult-born DG neurons have altered MF plasticity with CA3 remains unclear. Alterations in the fraction of newborn neurons that form atypical MF synapses could represent a mechanism by which experience and neurogenesis fine tune hippocampal activity.

Our results suggest that targeted cognitive enrichment training may be especially beneficial to individuals that have suffered from ELS. A potential therapeutic avenue is playing video action games, which significantly improves spatial reasoning and memory in humans. For instance, 3D video game players outperformed non-players on hippocampal-mediated memory functions such as mental rotation and spatial visualization tasks (Green and Bavelier, 2003; Uttal et al., 2013; Clemenson and Stark, 2015). Moreover, neuroimaging studies confirm that video game training increases gray matter in the hippocampus and prefrontal cortex (Kühn et al., 2014). Future studies, however, are needed to determine whether accessible interventions like 3D video games can mitigate the long-term cognitive and hippocampal structural deficits induced by early life adversity.

**FIGURE 5:**
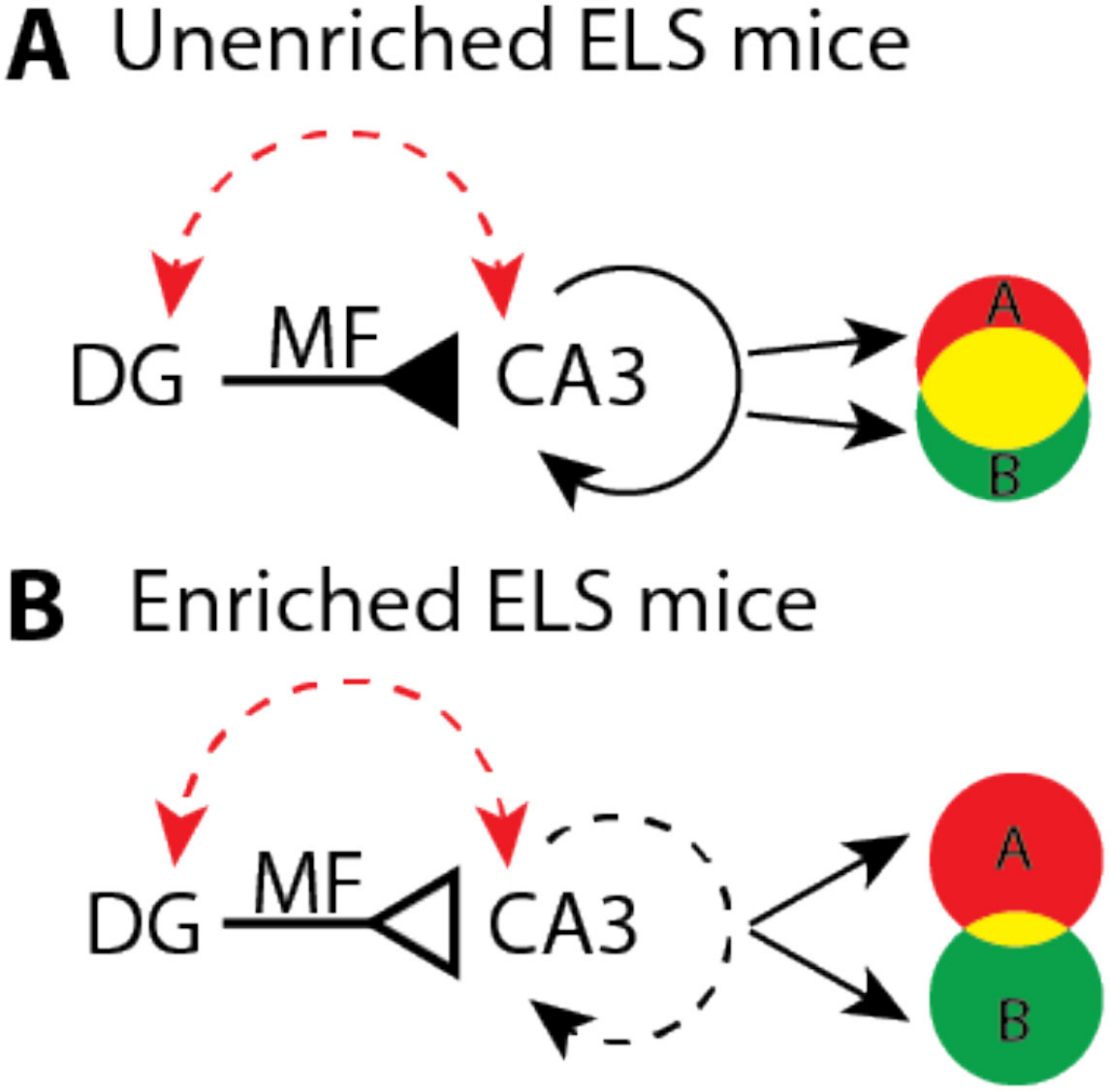
Hypothetical model that illustrates how enrichment could improve pattern separation in aged early-life stress (ELS) mice. **(A)** Age and stress (red dashed arrow) increase the excitability of dentate gyrus (DG) and CA3 which may cause mossy fiber (MF) input (solid inverted arrow) to drive dense recurrent activity (solid circular arrow). These dynamics could produce large overlaps (yellow shading) between context specific representations (A and B). **(B)** In enriched mice, age and stress may still lead to high levels of DG and CA3 excitability. However, a 27 percent reduction in the number of atypical MF synapses, per CA3 neuron, (open inverted arrow) could help to restore pattern separation by driving sparser recurrent activity (dashed circular arrow) that orthogonalizes (less yellow shading) context specific representations (A and B).

Our findings suggest a potential mechanism to restore spatial learning and hippocampal function in aged ELS mice. Studies show that age and ELS increase DG and CA3 excitability (Barnes and McNaughton, 1980; Wilson et al., 2005; Patrylo et al., 2007; Simkin et al., 2015; Villanueva-Castillo et al., 2017); changes that would increase population activity and impair the ability of the hippocampus to pattern separate (Barnes et al., 1997; Tanila et al., 1997a, 1997b; Redish et al., 1998; Wilson et al., 2005; Jinde et al., 2012; Figure 5A). Our cognitive enrichment causes a 27% decrease in the number of atypical MF synapses which could counteract high excitability by reducing the number of MF synapses. The resulting increase in sparse activity would favor pattern separation and improve spatial learning, as distinct contexts would have more orthogonal CA3 and CA1 representations (McNaughton and Morris, 1987; McHugh et al., 2007; Figure 5B).

## Materials and methods

### Experimental animals

For the longitudinal study we used Thy1-GCaMP6f-GP5.17 (Jackson) mice due to the absence of reporter expression in the CA3. Upon reaching early-adulthood (2.5 months) GCaMP6f^+^ litter-mates were double-housed (after ELS) so that each cage had a mouse that ran ET and CT. This consisted of four cages of females (*n* = 8) and four cages of males (*n* = 8) for a total of (*n* = 16). Due to the longevity of this study, three males and one female mouse died of natural causes before reaching the final 20 months timepoint. After removal of the GCaMP6f^+^ mice, there remained six male GCamP6f^–^ littermates (three cages). These were grouped with age typically reared (TR) age-matched C57BL/6J male mice purchased from Jackson (*n* = 6) for the ELS vs. TR experiment (Figures 2E, F).

### Early-life stress and cognitive enrichment

The experiments conducted were in accordance with the guidelines set by the Institutional Animal Care and Use Committee (IACUC) at the University of California, Irvine. We used the ELS protocol (developed in the Baram lab) whereby mouse litters receive limited bedding and nesting (LBN) conditions from postnatal days 2–10 in their rearing cages to modify maternal behavior during early development, (Figures 1A, B). Briefly, on postnatal day 2, we replaced the normal bedding (∼6 L) with a fitted metal grate (large enough to allow dropping to collect underneath) and two nestlets so that the dam could make a rudimentary nest. On postnatal day 10, we restored the normal bedding conditions. The control group (3 L), referred to as typically reared (TR), remained in their normal cages. After reaching adulthood (3.5 months), we ran a subset of the ELS mice (*N* = 16) on either enrichment (ET) or control track (CT) for ten weeks (3 × 30 min sessions/week), (Gattas et al., 2022; Figure 1C). Briefly, the enrichment setup consisted of two juxtaposed square tracks: one containing obstacles, while the other had simple ramps with one-way doors located at diagonal corners (Supplementary Figures 1A, B). Initially, we trained both groups of mice (2 weeks) to run laps around the track loaded with simple ramps (12) while receiving a single milk reward dispensed from a mounted lick tube triggered at the conclusion of each lap. In the next phase, we introduced complex obstacles only to the ET group, which continued for 8 weeks (Figure 1B).

### Object location memory and novel object recognition

For each time point, we conducted a set of OLM (first week) and NOR (second week) behaviors over a 2 weeks period (10 days total). The first 3 days of each week served as habituation sessions, during which animals explored empty square boxes (10 × 9 inches) for 10 min. On the training day (fourth day), mice were exposed to two identical objects for 10 min. On the test day (fifth day), one object was relocated to a new position (OLM, week 1, Figure 2A, top) or replaced with a novel object in the same position (NOR, week 2, Figure 2B, top). We used the same context box but different objects for each round of OLM and NOR to avoid object familiarity-related confounds. Boxes had unique markings on two of the walls (vertical and horizontal stripes) so that mice could easily associate the position of the objects with the box (Supplementary Figures 2A, B). All behaviors were recorded with an overhead camera using infrared emitters for low-light conditions.

### Y-maze

The Y-maze setup had three identical arms (3.5 inches wide and 10.5 inches long) with transparent walls and was surrounded by distinct external cues (Figure 2C). We always placed mice in the start arm at the beginning of each session. On training day (the first exposure to the Y-maze), we barricaded the to-be novel arm (opaque blocker) so that mice could only explore the two open arms (start and familiar) for 10 min. On test day (24 h later), we removed the blocker so that mice were free to explore all three arms for 5 min. A single top-view camera captured training and testing session videos.

### Behavioral analysis

The behavior videos were analyzed manually using BORIS software (Friard and Gamba, 2016) to count the duration each animal spent within 2 cm of each object (OLM/NOR) and moving inside the novel arm (spatial Y-maze). Memory performance in each behavior test was measured using discrimination ratio (DR), calculated as the ratio of time spent near novel conditions (object in a novel location for OLM or novel object for NOR or novel arm in spatial Y-maze) to time spent near familiar conditions (object in a familiar location for OLM or familiar object for NOR or familiar arm in spatial Y-maze).

### Immunofluorescence staining

Following isoflurane anesthesia, mice were transcardially perfused with cold phosphate buffer solution (PBS), followed by 4% paraformaldehyde (PFA), and the extracted brain was stored in 4% PFA at 4 °C. Before slicing, brains were transferred to 30% sucrose/PBS solution and stored at 4 °C for cryoprotection. Brains were sectioned at 40 μm using a cryostat (Thermo Scientific HM525 NX) at −20 °C, and each section was stored in well plates containing cryoprotectant solution.

Anatomical targeting: Dorsal hippocampal sections were selected at approximately −1.8 mm from bregma (anterior-posterior coordinate) according to the Paxinos and Watson mouse brain atlas to visualize mossy fiber (MF) synapses in the distal CA3 region. This anatomical level was chosen to ensure regional consistency, as synaptogenesis is region-specific within hippocampal subfields, particularly in the septal versus temporal hippocampus.

Immunohistochemistry protocol: Initial attempts using traditional PSD-95 and presynaptic antibodies yielded poor results with low signal and poor colocalization. Subsequent optimization using nanobodies showed dramatic improvements. First, we incubated sections for 2 h (at room temperature) in PBS blocking buffer containing 5% normal goat serum (NGS) and 0.3% Triton X-100. Sections were then incubated with nanobodies (purchased from NanoTag Biotechnologies) that contained the appropriate combination of either anti-PSD-95 nanobody (FluoTag^®^-X2 anti-PSD95, Cat No: N3702-AF647-L, 2 nM, Alexa647), anti-synaptotagmin 1 nanobody (FluoTag^®^-X2 anti-Synaptotagmin 1, Cat No: N2302-AF568-L, 0.2 nM, AZDye568) or VGLUT1 nanobody (FluoTag^®^-X2 anti-VGLUT1, Cat No: N1602-AF568-L, 1 nM, AZDye568). Incubation was performed with shaking at 4 °C for 24 h in PBS containing 0.3% Triton X-100. Sections were washed four times for 10 min each alternating between PBS and TBS. At these low concentrations, fluorescent signals were more prominent in the stratum pyramidale (SP) layer compared to the stratum lucidum (SL) layer. Sections were mounted onto slides with media containing DAPI (Invitrogen SlowFade Glass Soft-set Antifade Mountant with DAPI, catalogue #S36917).

### Gross morphological analysis

We took images of the entire dorsal hippocampus with a Keyence BZ-X1810 widefield fluorescence microscope with a 20x objective lens with DAPI and GCaMP filter cubes to visualize cell bodies and endogenous GCaMP6 signal. Images were loaded in Zeiss Zen software so that we could manually trace hippocampal subfields using the active contour tool. Following each outline, we took reported areas and normalized them to the total hippocampal area. To determine number of CA3 neuron we counted the number of easily identifiable large dark circles in the tiled images (described below). All tracing and counting was done by a double-blind observer.

### Mossy fiber synaptic analysis

Confocal imaging: Tiled images of the distal CA3 bend were captured using an LSM 900 microscope equipped with Airyscan 2 and a 63X objective lens. PSD-95 and synaptotagmin-1 were excited using 653 and 568 nm diode lasers, respectively. Images were over-sampled (2x) to facilitate super-resolution post-processing with Zeiss’ Super Resolution Airyscan mode (2D, auto). No additional deconvolution was applied beyond the Airyscan super-resolution processing. Images were manually aligned using Imaris Stitcher, creating two composite panels per mouse (approximately 20 stitched images for each z-plane panel) selected systematically from the dorsal and ventral aspects of the CA3 bend. These panels were roughly 8,000 × 10,000 pixels (∼350 × 420 μm), 16-bit TIFF files, featuring red (PSD-95) and green (synaptotagmin-1) channels. Imaging parameters including laser power (2.3% for 640 nm and 3.0% for 561 nm), gain, and offset were kept constant across all samples to ensure quantitative comparisons. As expected, nanobody penetration was lowest in the middle of the slice so for each mouse we constructed two composite panels at 10 and 30 μm positions within the 40 μm slice. We used the same settings for non-tiled images with the following additions: laser 405 nm at 1.5% and laser 488 nm power at 1.3%. Z-stacks consisted of 15 optical sections centered at 10 μm with a 1 μm step size. Reconstructed 3D images of synapses surrounding the cell body are max Z projections of all 15 slices.

Quantitative analysis: For subsequent analysis on the tiled images, FIJI was used to crop images focusing on the pyramidal cell bodies of the SP layer. These cropped, composite images served as input for the MF synapse detection algorithm. Each channel was independently thresholded based on fluorescence intensity to create binary masks of equal size. Threshold values of 515 for PSD-95 and 350 for Synaptotagmin were selected because they captured all identifiable clusters while excluding apparent noise, with similar mask sizes observed with threshold variations within a 5% range. The masks were combined using a pixel-wise AND operation and applied to the original 2-channel image, retaining only pixel values above the respective thresholds, with all other pixels set to 0 (black). Clusters smaller than 150 pixels were removed from subsequent analysis to eliminate imaging artifacts, so only clusters with an area exceeding 75% of 0.37 μm^2^ (0.27 μm^2^) were included. Large images were processed in parallel using smaller blocks of 500 × 500 pixels, with a flood-fill algorithm applied to every fourth pixel (provided it was a non-zero value) to detect cluster masks. Visual inspections were performed on two images by manual counting to validate the automated detection, showing 97% agreement between automated and manual counts. Duplicate detections at block edges were filtered to ensure a unique set of clusters. Mossy fiber bouton density was quantified as the number of synaptic clusters per CA3 neuron within the pyramidal cell layer, normalized across the two composite panels per mouse. All image acquisition and quantitative analyses were performed by investigators blinded to experimental groups. The analysis code is freely available online at GitHub.^1^

### Statistical analysis

Behavioral tests were analyzed using repeated measures and two-way ANOVA designs (α = 0.05). Object location memory (OLM) and novel object recognition (NOR) were analyzed using three-way repeated measures ANOVA (Age × Group × Sex) with *n* = 16 mice total (ELS + ET: 4M and 4F, *n* = 8; ELS + CT: 4M and 4F, *n* = 8). For OLM, only the Group main effect was statistically significant [F(1,12) = 8.80, *p* = 0.012], while all other main effects and interactions were non-significant: Age [F(1,12) = 1.69, *p* = 0.218], Sex [F(1,12) = 0.97, *p* = 0.345], Age × Group [F(1,12) = 0.16, *p* = 0.700], Age × Sex [F(1,12) = 0.081, *p* = 0.780], Group × Sex [F(1,12) = 0.0004, *p* = 0.984], and Age × Group × Sex [F(1,12) = 0.33, *p* = 0.579]. For NOR, no main effects or interactions reached statistical significance: Age [F(1,12) = 2.93, *p* = 0.113], Group [F(1,12) = 0.074, *p* = 0.791], Sex [F(1,12) = 0.11, *p* = 0.744], Age × Group [F(1,12) = 1.66, *p* = 0.222], Age × Sex [F(1,12) = 0.53, *p* = 0.479], Group × Sex [F(1,12) = 4.48, *p* = 0.056], and Age × Group × Sex [F(1,12) = 0.55, *p* = 0.472]. Object exploration time (OLM and NOR combined) was analyzed using a two-way ANOVA (Test Type × Group) with the same sample sizes. Only the Test Type main effect was statistically significant [F(2,88) = 30.86, *p* < 0.0001], while Group [F(1,88) = 0.886, *p* = 0.349] and Test Type × Group interaction [F(2,88) = 0.101, *p* = 0.904] were non-significant. Y-maze data were analyzed using two-way ANOVA (Group × Sex) with *n* = 12 mice total (ELS + ET: 2M and 3F, *n* = 5; ELS + CT: 3M and 4F, *n* = 7). Only the Group main effect was statistically significant [F(1,8) = 19.89, *p* = 0.002], while Sex [F(1,8) = 1.81, *p* = 0.215] and Group × Sex interaction [F(1,8) = 0.81, *p* = 0.393] were non-significant.

Synaptic analyses were conducted using two-way ANOVA (Group × Sex, α = 0.05) with *n* = 12 mice total (ELS + ET: 2M and 3F, *n* = 5; ELS + CT: 3M and 4F, *n* = 7). A significant Group main effect was found for cluster count [F(1,8) = 6.07, *p* = 0.039], with Sex [F(1,8) = 0.043, *p* = 0.841] and Group × Sex interaction [F(1,8) = 0.073, *p* = 0.794] being non-significant. For cluster size, no main effects or interactions reached statistical significance: Group [F(1,8) = 4.36, *p* = 0.070], Sex [F(1,8) = 0.0009, *p* = 0.976], and Group × Sex interaction [F(1,8) = 0.42, *p* = 0.535].

Gross morphological analyses were conducted using two-way ANOVA (Group × Sex, α = 0.05) with *n* = 12 mice total (ELS + ET: 2M and 3F, *n* = 5; ELS + CT: 3M and 4F, *n* = 7). All hippocampal morphology measures showed non-significant results including CA3 neuron density [Group: F(1,8) = 1.734, *p* = 0.224; Sex: F(1,8) = 0.026, *p* = 0.876; Group × Sex: F(1,8) = 0.012, *p* = 0.917], hilus area [Group: F(1,8) = 0.318, *p* = 0.589; Sex: F(1,8) = 0.268, *p* = 0.619; Group × Sex: F(1,8) = 0.117, *p* = 0.741], mossy fiber area [Group: F(1,8) = 0.596, *p* = 0.462; Sex: F(1,8) = 4.265, *p* = 0.073; Group × Sex: F(1,8) = 0.144, *p* = 0.714], and dentate gyrus area [Group: F(1,8) = 0.024, *p* = 0.881; Sex: F(1,8) = 4.056, *p* = 0.079; Group × Sex: F(1,8) = 0.098, *p* = 0.762]. Sex effects approached significance for both mossy fiber area (*p* = 0.073) and dentate gyrus area (*p* = 0.079).

Summary of significant findings: Cognitive enrichment training (ET) significantly improved spatial memory performance in ELS mice, as evidenced by significant Group effects in both OLM (*p* = 0.012) and Y-maze (*p* = 0.002) tasks, but not in the hippocampus-independent NOR task. At the synaptic level, ET training significantly reduced the number of atypical mossy fiber synaptic clusters surrounding CA3 cell bodies (*p* = 0.039), while gross hippocampal morphology remained unchanged. These findings suggest that cognitive enrichment rescues ELS-induced spatial memory deficits through selective modifications of synaptic connectivity rather than gross structural changes.

## Data Availability

The original contributions presented in this study are included in this article/Supplementary material, further inquiries can be directed to the corresponding author.

## Footnotes

1
https://github.com/bainro/MF-synapse-analysis
