# Supplementary material for: Cognitive enrichment improves spatial memory and alters hippocampal synaptic connectivity in a mouse model for early-life stress
Source: Front Cell Neurosci. 2025 Oct 17;19:1646883. doi: 10.3389/fncel.2025.1646883 (PMC12575294; doi:10.3389/fncel.2025.1646883)
Supplement: Supplementary file 2 [file Presentation_2.pptx]

## Slide 1
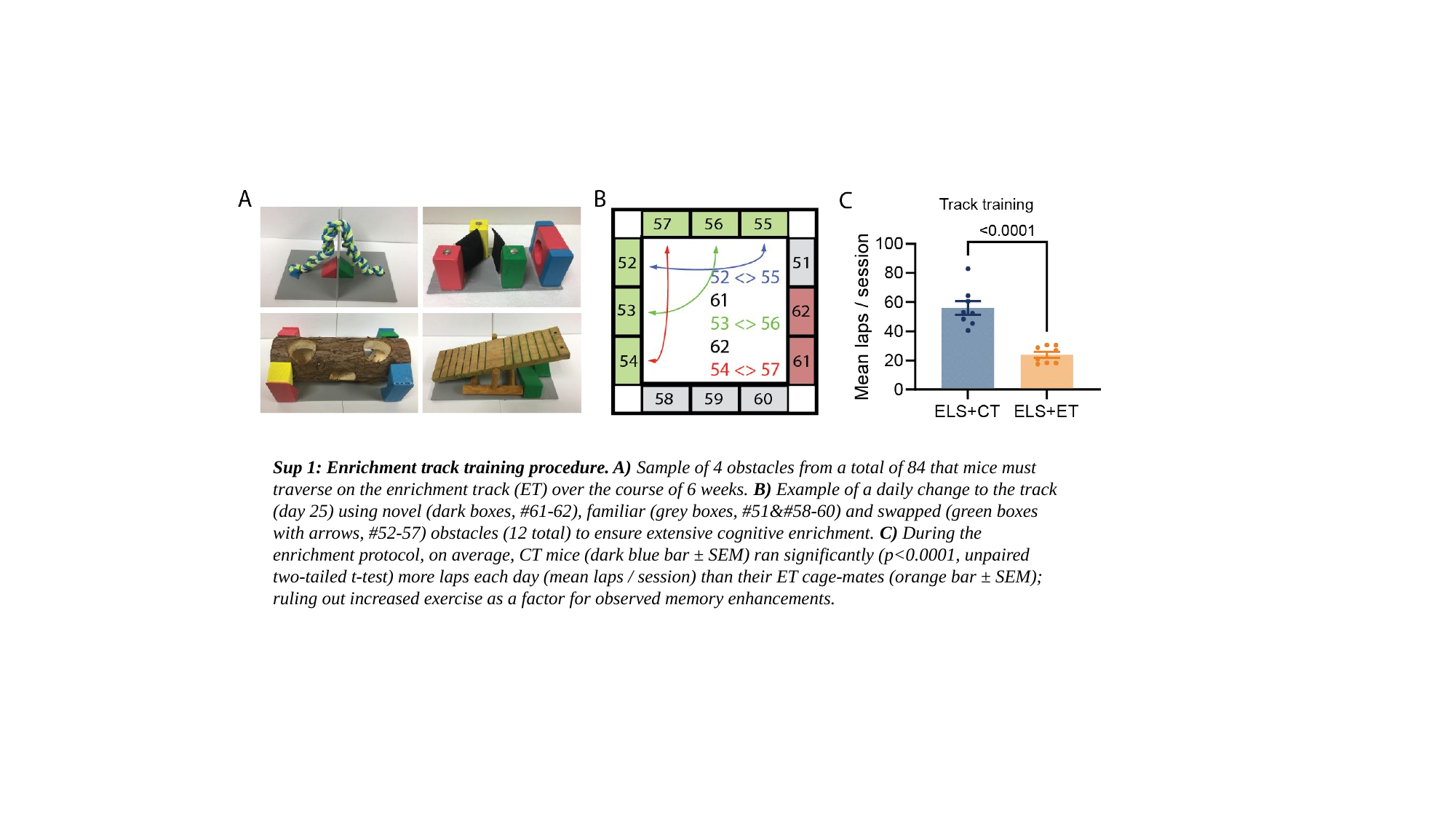

Sup 1: Enrichment track training procedure. A) Sample of 4 obstacles from a total of 84 that mice must traverse on the enrichment track (ET) over the course of 6 weeks. B) Example of a daily change to the track (day 25) using novel (dark boxes, #61-62), familiar (grey boxes, #51&#58-60) and swapped (green boxes with arrows, #52-57) obstacles (12 total) to ensure extensive cognitive enrichment. C) During the enrichment protocol, on average, CT mice (dark blue bar ± SEM) ran significantly (p<0.0001, unpaired two-tailed t-test) more laps each day (mean laps / session) than their ET cage-mates (orange bar ± SEM); ruling out increased exercise as a factor for observed memory enhancements.

## Slide 2
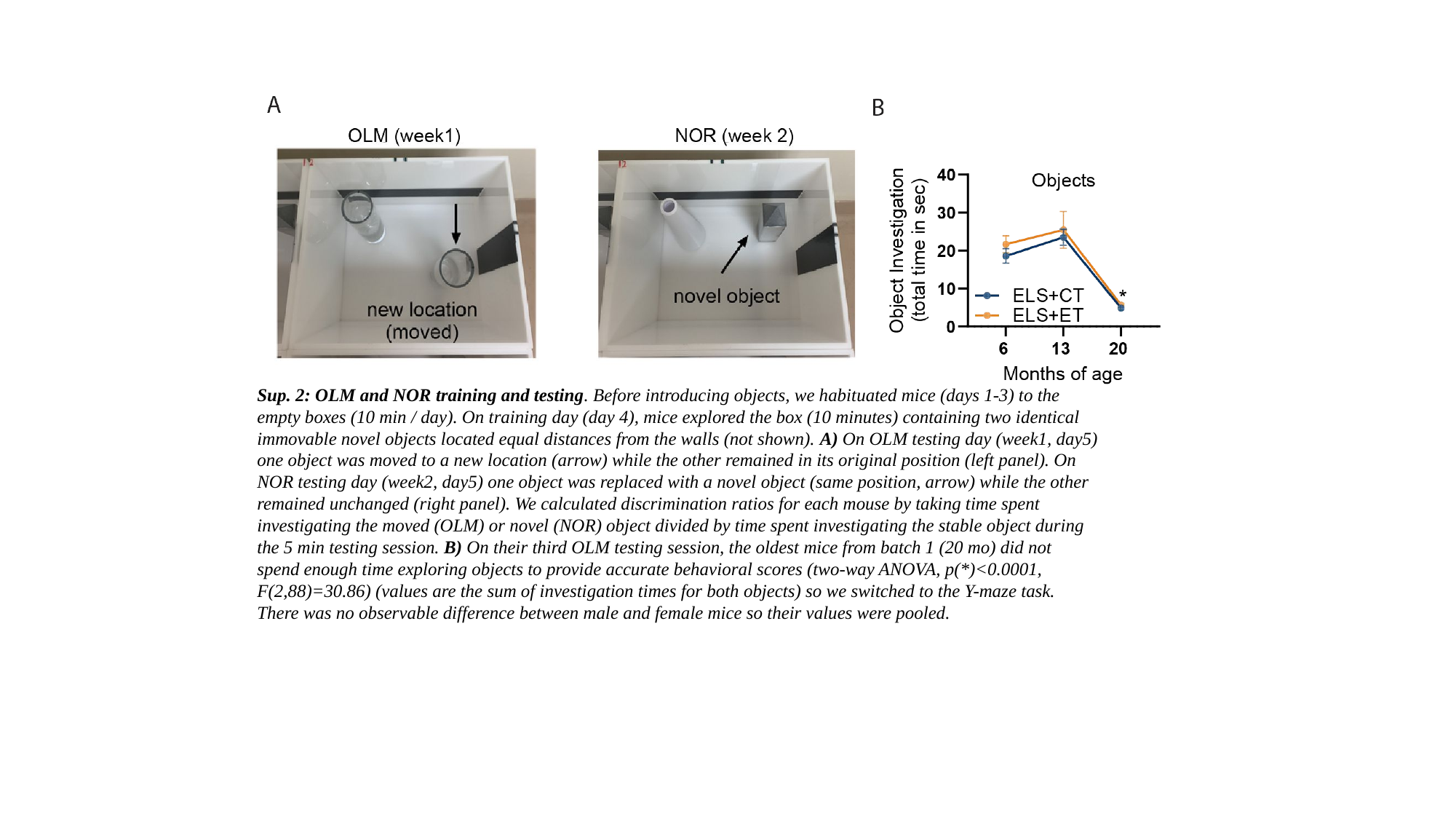

Sup. 2: OLM and NOR training and testing. Before introducing objects, we habituated mice (days 1-3) to the empty boxes (10 min / day). On training day (day 4), mice explored the box (10 minutes) containing two identical immovable novel objects located equal distances from the walls (not shown). A) On OLM testing day (week1, day5) one object was moved to a new location (arrow) while the other remained in its original position (left panel). On NOR testing day (week2, day5) one object was replaced with a novel object (same position, arrow) while the other remained unchanged (right panel). We calculated discrimination ratios for each mouse by taking time spent investigating the moved (OLM) or novel (NOR) object divided by time spent investigating the stable object during the 5 min testing session. B) On their third OLM testing session, the oldest mice from batch 1 (20 mo) did not spend enough time exploring objects to provide accurate behavioral scores (two-way ANOVA, p(*)<0.0001, F(2,88)=30.86) (values are the sum of investigation times for both objects) so we switched to the Y-maze task. There was no observable difference between male and female mice so their values were pooled.

## Slide 3
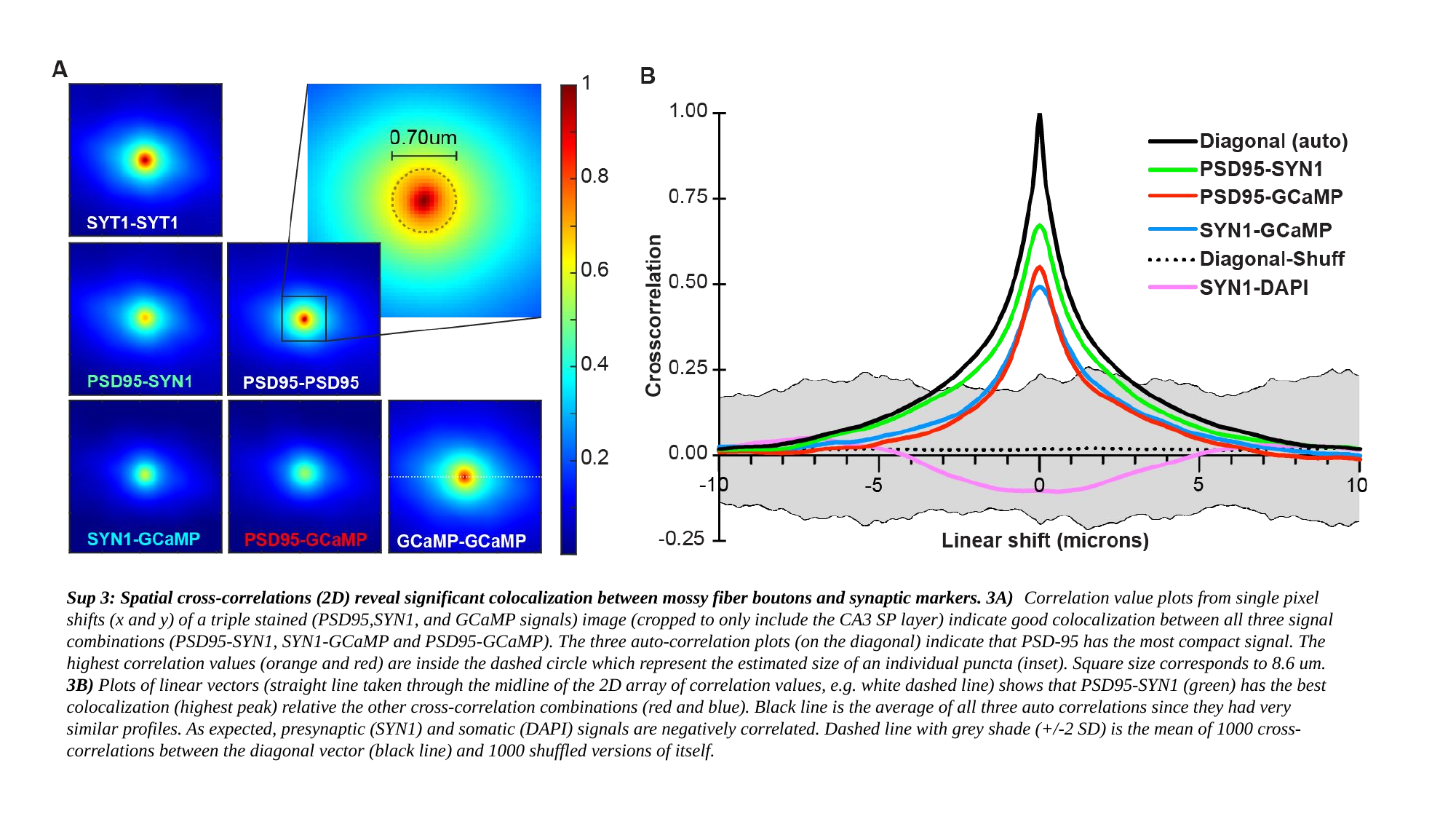

Sup 3: Spatial cross-correlations (2D) reveal significant colocalization between mossy fiber boutons and synaptic markers. 3A) Correlation value plots from single pixel shifts (x and y) of a triple stained (PSD95,SYN1, and GCaMP signals) image (cropped to only include the CA3 SP layer) indicate good colocalization between all three signal combinations (PSD95-SYN1, SYN1-GCaMP and PSD95-GCaMP). The three auto-correlation plots (on the diagonal) indicate that PSD-95 has the most compact signal. The highest correlation values (orange and red) are inside the dashed circle which represent the estimated size of an individual puncta (inset). Square size corresponds to 8.6 um. 3B) Plots of linear vectors (straight line taken through the midline of the 2D array of correlation values, e.g. white dashed line) shows that PSD95-SYN1 (green) has the best colocalization (highest peak) relative the other cross-correlation combinations (red and blue). Black line is the average of all three auto correlations since they had very similar profiles. As expected, presynaptic (SYN1) and somatic (DAPI) signals are negatively correlated. Dashed line with grey shade (+/-2 SD) is the mean of 1000 cross-correlations between the diagonal vector (black line) and 1000 shuffled versions of itself.

## Slide 4
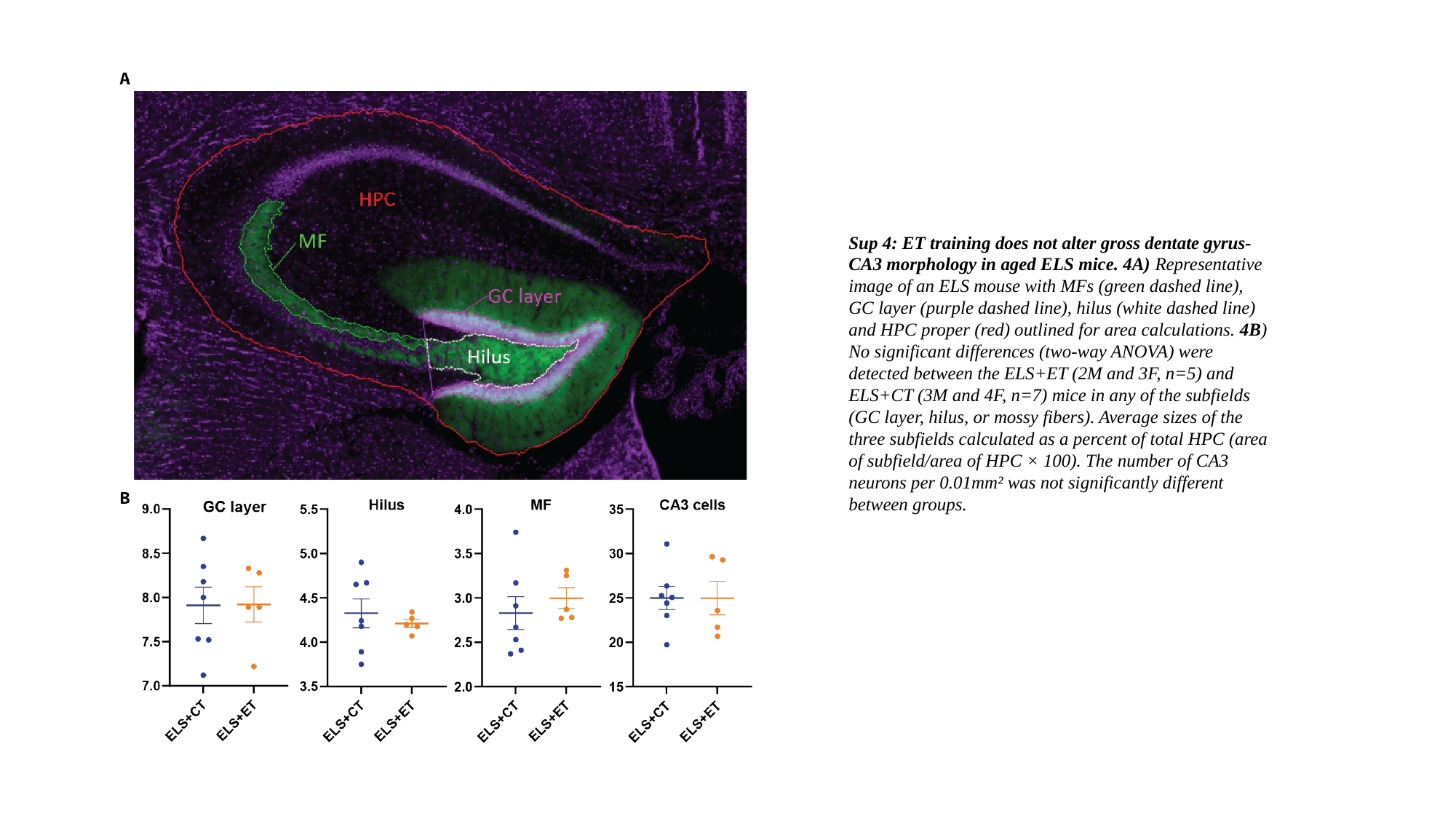

Sup 4: ET training does not alter gross dentate gyrus-CA3 morphology in aged ELS mice. 4A) Representative image of an ELS mouse with MFs (green dashed line), GC layer (purple dashed line), hilus (white dashed line) and HPC proper (red) outlined for area calculations. 4B) No significant differences (two-way ANOVA) were detected between the ELS+ET (2M and 3F, n=5) and ELS+CT (3M and 4F, n=7) mice in any of the subfields (GC layer, hilus, or mossy fibers). Average sizes of the three subfields calculated as a percent of total HPC (area of subfield/area of HPC × 100). The number of CA3 neurons per 0.01mm² was not significantly different between groups.

## Slide 5
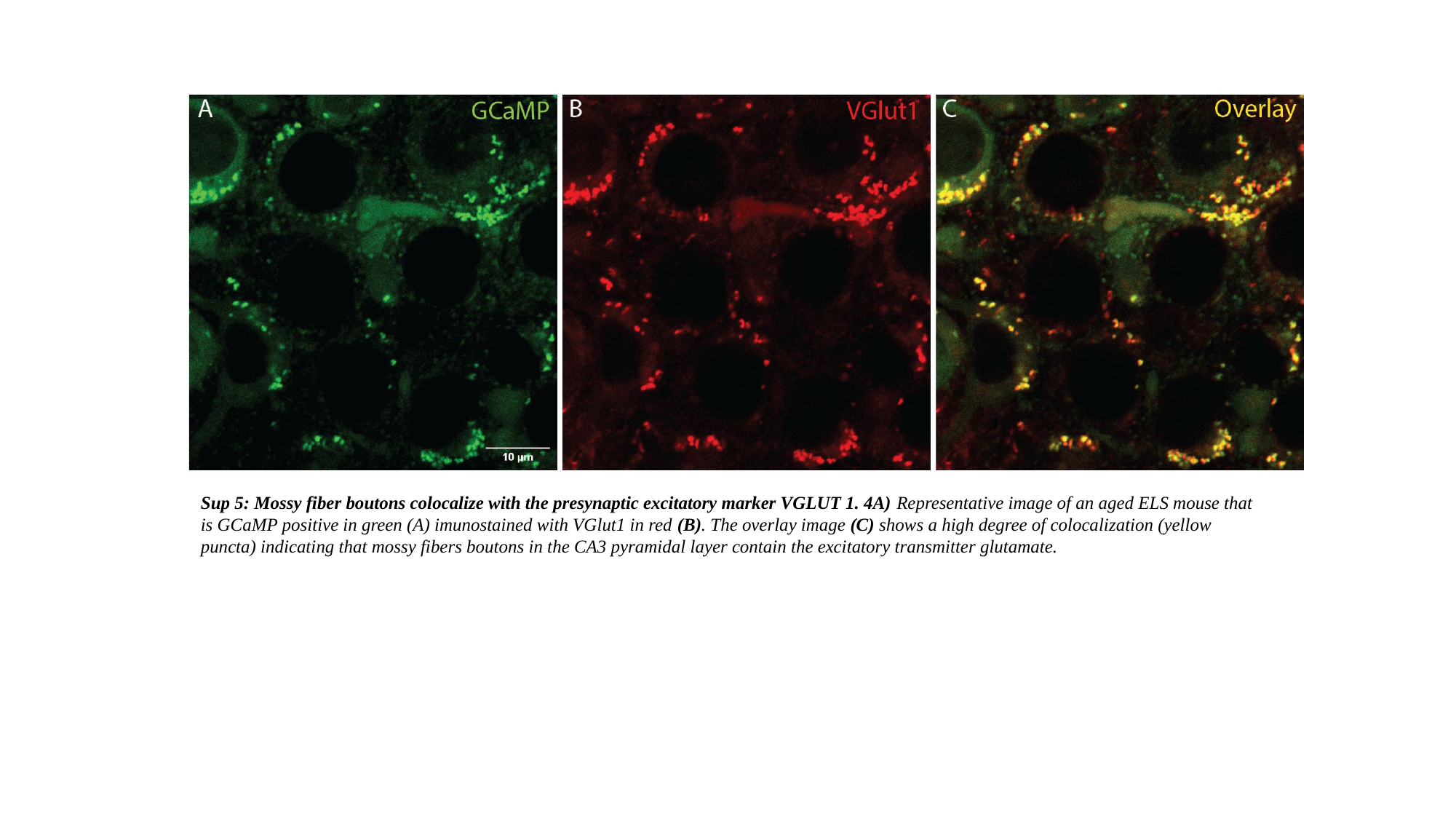

Sup 5: Mossy fiber boutons colocalize with the presynaptic excitatory marker VGLUT 1. 4A) Representative image of an aged ELS mouse that is GCaMP positive in green (A) imunostained with VGlut1 in red (B). The overlay image (C) shows a high degree of colocalization (yellow puncta) indicating that mossy fibers boutons in the CA3 pyramidal layer contain the excitatory transmitter glutamate.
